# A Financing Strategy to Expand Surgical Health Care

**DOI:** 10.9745/GHSP-D-21-00295

**Published:** 2023-06-21

**Authors:** Desmond T. Jumbam, Che L. Reddy, John G. Meara, Emmanuel M. Makasa, Rifat Atun

**Affiliations:** a Department of Policy and Advocacy, Operation Smile, Virginia Beach, VA, USA.; b Program in Global Surgery and Social Change, Department of Global Health and Social Medicine, Harvard Medical School, Boston, MA, USA.; c Department of Plastic and Oral Surgery, Boston Children’s Hospital, Boston, MA, USA.; d Health Systems Innovation Lab, Department of Global Health and Population, Harvard T.H. Chan School of Public Health, Harvard University, Boston, MA, USA.; e Wits Centre of Surgical Care for Primary Health and Sustainable Development, School of Clinical Medicine, University of Witwatersrand, Johannesburg, South Africa.; f Department of Global Health and Social Medicine, Harvard Medical School, Boston, MA, USA.; g Department of Global Health and Population, Harvard T.H. Chan School of Public Health, Boston, MA, USA.

## Abstract

Despite an evolving need to provide surgical health care globally, few health systems, particularly in low-income and middle-income countries (LMICs), can sufficiently provide such care. The vast majority of the world’s people—an estimated 5 billion—are unable to access safe and affordable surgical health care when they need it. This is a significant concern for global public health because the demand for these services is rising with the epidemiological and demographic transitions occurring worldwide. A principal driver of weak surgical health care services is a lack of adequate health system financing for surgical health care. This article examines the financing of surgical health care by analyzing global trends in health system financing, approaches to expand fiscal space for health, and empirical perspectives on the design, introduction, and scale-up of policies to improve surgical systems. We describe a surgical health care financing strategy, together with broader political and economic considerations, to provide policy recommendations to fund the expansion of surgical health care and an essential surgical package as part of universal health coverage in LMICs.

## Abstract (precis)

The authors propose a strategy to guide the financing of surgical health care services as part of national health care policies in LMICs.

## Teaser

**Key Findings:**

- Despite the strategic efforts taken by numerous low- and middle-income countries to develop national surgical, obstetric, and anesthesia plans (NSOAPs) to address surgical needs at a population level, financing these policies remains a challenge.
- To address the surgical financing challenge, it is necessary to examine the fiscal space options for financing such policies from the very beginning of policy development and throughout the development process.

**Key Implications:**

- The proposed surgical health care financing strategy (SHFS) uses an analytic iterative process to identify potential sources of funding, quantify the investment, and mobilize political support for surgical systems at a national level.
- The SHFS could also help to promote responsibility, accountability, and transparency about financial commitments around funding NSOAPs and other policies designed to improve surgical health care through the implementation of an essential surgical package via universal health coverage.

## INTRODUCTION

While the provision of surgical health care services is core to any health system and the attainment of universal health coverage (UHC), few low- and middle-income countries (LMICs) can sufficiently provide such health care.^1^ The vast majority of the world’s population—an estimated 5 billion people, who mostly live in LMICs—does not have access to high-quality and affordable surgical health care services when needed.^2^ Inadequate financing for surgical health care is a principal driver of this health system shortfall. Failure to invest appropriately in surgical ecosystems in LMICs leads to substantial health and economic loss.^3^ Functioning surgical ecosystems are needed to counter the rising surgical demand due to the epidemiological transition^4^ and global health threats, including climate change^5^ and pandemics such as COVID-19.^6^^,^^7^ The surgical ecosystem—the people, infrastructure, and processes involved in providing all surgical subdisciplines, anesthesia, and intensive care—will continue to be a critical ingredient of health systems. However, sustainable health system investment in surgical health care is lacking.^1^^,^^8^^,^^9^

This article examines options for financing expanded surgical health care services in LMICs as part of broader efforts to achieve UHC. The study analyzes trends in health systems financing, approaches to expand fiscal space for health, and empirical perspectives on the design and scale-up of policies to improve surgical systems to develop a strategy to fund the expansion of surgical health care in LMICs. The article is organized into 3 parts. Part 1 examines the critical contextual elements needed to understand the financing of surgical health care in LMICs. Specifically, it explores the evolving global health priority of surgical health care, current policy interventions to improve surgical health care, and the current state of funding surgical health care within broader trends in LMIC health system financing. In Part 2, we discuss the key features, components, and process of a surgical health care financing strategy (SHFS). We conclude in Part 3 by proposing a unifying framework with policy considerations for how policymakers could adopt the SHFS approach to fund the expansion of surgical health care as part of ongoing efforts to achieve UHC.

We examine options for financing expanded surgical health care services in LMICs as part of broader efforts to achieve UHC.

## PART 1: THE CONTEXT OF FINANCING SURGICAL HEALTH CARE IN LMICS

### Surgical Health Care Need

In LMICs, contemporary health system challenges center around socioeconomic, demographic, and epidemiological transitions that are leading to aging populations and rapidly increasing burdens of noncommunicable diseases (NCDs) and injuries.^10^ These transitions are expected to increase the need for surgical health care.^1^ The Lancet Commission on Global Surgery reported that between 28%–32% of the global burden of disease (based on the 2010 Global Burden of Disease study from 1990 to 2010) includes conditions that require surgical health care services (operations, procedures, and specialist consultations).^11^

Failure to invest in health systems to deliver quality surgical health care services could result in substantial disability, mortality,^12^ and macroeconomic loss^13^—particularly for LMICs—which could be averted.^1^ It is estimated that 16.9 million lives are lost from conditions requiring surgical health care each year.^11^ Where available, poor quality surgical health care is also a major cause of global mortality; postoperative mortality could be responsible for at least 4.2 million deaths each year, making it the third highest cause of global mortality.^14^ The Lancet Commission on Global Surgery reported that between 2015 and 2030, untreated surgical conditions could result in the cumulative loss of US$20.7 trillion in the global economy (1.3% of projected economic output) with more than half (US$12.3 trillion) of all losses occurring in LMICs.^13^ It is also worth noting the trend that as country gross domestic product increases, so does greater public coverage for health care services, as was the case in Malaysia, and that the introduction of national health insurance schemes also leads to greater public coverage, as in Ghana and Mexico.

Governments have a vital responsibility to ensure that their societies have access to quality surgical health care services as a way of securing the much-needed human capital^15^ for national development and also to fulfill their international commitments. Examples of such commitments relevant to surgical health care include the Sustainable Development Goals,^16^ United Nations high-level political declaration on UHC,^17^ World Health Assembly resolution 68.15 on strengthening emergency and essential surgical care as a component of UHC,^18^ and World Health Assembly decision 70/22 to monitor member state progress on WHA 68.15.^19^ At a national level, surgical health care should be an essential health care service, provided as part of UHC.^20^ If civil society mobilizes to support surgical health care in the form of activist interest groups (like the Treatment Action Campaign in the case of HIV/AIDS care^21^), citizens could exert substantial political pressure on their governments to provide surgical health care as part of comprehensive UHC packages.

### Policy Interventions to Improve Surgical Health Care and Define an Essential Surgical Package as Part of UHC

From a health policy standpoint, surgical health care has previously been of only low to moderate importance in LMICs.^22^^,^^23^ However, in response to scientific evidence on the burden of surgical disease and advocacy efforts by various global surgery groups,^24^ many LMICs are developing policy strategies to improve surgical health care in the form of national surgical, obstetric, and anesthesia plans (NSOAPs).^1^^,^^25^^,^^26^ Critically, in the context of UHC, these plans provide a process for countries to design an essential surgical package and articulate strategies for scale-up as part of UHC.^20^ NSOAPs are flexible policy instruments designed for integration into national health strategic plans and implementation through state and nonstate actors.^27^ The NSOAP process is as important as the end result. The plans foster the participation of diverse stakeholder groups to formulate collective positions on how to improve surgical health care, given contextual realities, sociopolitical norms, and economic prospects.^1^^,^^28^ The United Nations Institute for Training and Research has developed and published an NSOAP manual to guide countries in the planning process.

NSOAPs provide a process for countries to design an essential surgical package and articulate strategies for scale-up as part of UHC.

NSOAPs have been adopted at national and regional levels. Ethiopia, Madagascar, Nigeria, Rwanda, Senegal, Tanzania, and Zambia have completed plans and have commenced with implementation.^26^^,^^29^^,^^30^ Pakistan was the first country in Asia to develop a national surgical policy, termed the National Vision for Surgical Care, which adopted a federal-provincial decentralized model. Several other countries across Africa, Asia, and South America are developing similar innovative country-specific plans through the NSOAP process or are formally committed to developing them through regional resolutions.^31^ In 2018, for example, the 16 member states of the Southern African Development Community passed a regional intergovernmental resolution to improve surgical health care at a national and regional level as priority work for UHC. One of the 5 components of this resolution is to develop NSOAPs.^32^ A similar approach was adopted by the Pacific Island nations. During the 13^th^ Pacific Health Ministers Meeting in 2019, the health ministers committed to developing and implementing NSOAPs as part of the regional effort to advance “the safe and affordable surgery agenda” and achieve UHC.^33^ Within the region, Cook Islands, Fiji, Palau, Tonga, and Vanuatu are developing NSOAPs. Despite these achievements in the level of attention and priority afforded to surgical health care at the government level, however, no country has committed substantial funding of their national health budgets towards implementation.

### Surgical Health Care in the Health Financing Landscape

#### Health Care Spending Is Rising Globally

Globally, on average, growth in health spending has outstripped that of the economy.^34^ From 1995 to 2014, total health spending per capita globally increased in real terms by 3.3% each year, compared to 3% in annual economic growth.^35^ The increase in health spending is observed across all country income groups but is highest for LMICs. Upper-middle-income countries, in particular, have the highest annual growth rates in spending per capita (5.5%), driven by increases in government financing.^36^ The global trend of rising health expenditure is expected to continue, given the many challenges faced by health systems.^37^^–^^39^ In LMICs, health care is 1 of the many development areas that need prioritization, leading to difficult and often highly politicized resource allocation decisions.

The level of economic development influences how health systems are financed.^40^ In low-income countries (LICs), health spending per capita is dependent on development assistance for health (DAH) and out-of-pocket (OOP) expenses. In 2014, DAH and OOP constituted more than 60% of health spending in most LICs.^35^ For countries in transition to middle-income status, health spending per capita is more reliant on domestic government spending and OOP and less DAH-dependent. In upper-middle-income countries and most high-income countries, the principal source of health spending is the domestic government.

How to sustainably finance health systems is a crucial challenge for LICs to address if they are to attain national and global health targets. Despite projections that show LICs to have the second-highest annualized rate of real growth in health spending of all income groups, health spending per capita is likely to remain low by 2030.^41^ Providing domestic funding for health and developing alternative mechanisms (e.g., innovative financing)^42^ will be critical to improving the sustainability of LIC health system financing, given the unpredictable nature of DAH, which has been decreasing since 2010.^43^^–^^45^

How to sustainably finance health systems is a crucial challenge for LICs to address if they are to attain national and global health targets.

#### Spending for Surgical Health Care Is Limited

In LMICs, resource allocation to surgical health care is inadequate.^1^^,^^8^^,^^12^ At the national level, financing comes from 3 sources: public, private (private insurance and OOP expenditure), and voluntary (nongovernmental and faith-based organizations). Global funding is in the form of DAH from bilateral organizations, philanthropic entities, and foundations.

At the national level, OOP is the dominant source of surgical health care expenditure in most LMICs.^8^ In terms of public expenditure, it is difficult to assess how much governments allocate to surgical health care; this is due to the difficulty of distinguishing between surgical and nonsurgical health expenditures in national health accounts,^46^ but reports suggest low levels of funding that are fragmented and inefficiently applied.^1^^,^^12^ At the global level, minimal DAH is allocated to surgical health care.^9^ Between 1995 and 2014, of the $423 billion DAH disbursed, 24% was allocated to HIV/AIDS, 15% to child health projects, 11% to maternal health projects, and 1.2% to NCDs^47^; NCDs were responsible for 73% of all deaths in 2017.^48^ According to the WHO, an estimated 86% of NCD deaths occur in LMICs.

The Lancet Commission on Global Surgery estimated a total cost of US$300–$400 billion to scale up surgical health care in LMICs.^1^ However, none of the countries to develop and cost NSOAPs (Supplement Table 1) have committed substantial amounts of additional funding to implementation. NSOAP implementation will require 0.1%–0.9% of gross domestic product per capita in these countries (Table 1).^31^ The significant differences in cost per gross domestic product per capita of implementing NSOAP reflect differences in country priorities. Nigeria, for example, includes the costs associated with scaling up UHC, which accounted for 56% of the total NSOAP compared to just 0.14% of the Rwandan NSOAP. Given inadequate levels of health system funding allocated to surgical health care, there is a need to adopt realistic strategies for its financing if it is to be accessible to all through UHC.

**TABLE 1.** Comparative Costing of NSOAPs in Low- and Middle-Income Countries^a^

**Country**	**ImplementationTime, Years**	**NSOAP TotalCost, US$**	**Current Health ExpenditurePer Capita, US$**	**NSOAP Cost/YearPer Capita, US$**
Zambia	5	314 million	1509.80	3.62
Tanzania	7	597 million	936.33	1.51
Rwanda	6	69 million	748.39	0.94
Nigeria	5	16 billion	1968.56	17.12

Abbreviations: NSOAP, national surgical, obstetric, and anesthesia plan; US$, U.S. dollars.

^a^Adapted from Jumbam et al.^31^

## PART 2: THE SURGICAL HEALTH CARE FINANCING STRATEGY

### A Strategy to Fund Surgical Health Care

**FIGURE 1:**
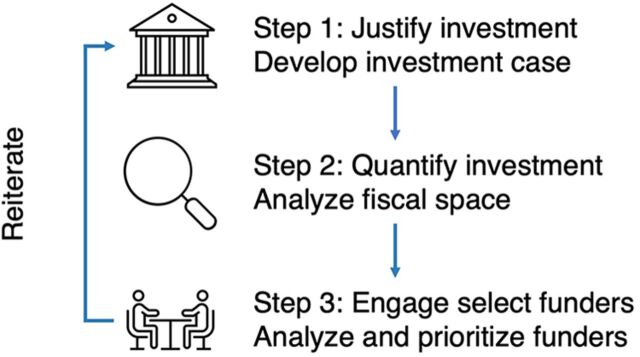
Steps of the Surgical Health Care Financing Strategy

Not all countries begin the NSOAP process (or any policy effort to improve surgical health care) by examining approaches to finance the development and implementation of the policy. Failing to make financial provisions for the NSOAP may lead to a plan that is not financially viable and hence not feasible. The SHFS (Figure 1) uses an analytic process to identify potential sources of funding, quantify the investment, and mobilize political support for surgical systems at a national level. It is an iterative process that occurs alongside policy instruments like NSOAPs that are designed to improve surgical health care and define an essential surgical package. We outline the 3 steps of the SHFS process below.

The SHFS uses an iterative, analytic process to identify potential funding, quantify the investment, and mobilize political support for surgical systems at a national level.

Ideally, the ministry of health (MOH) should lead the SHFS in close partnership with the ministry of finance (MOF) to determine from whom, when, how, and under what conditions funds will materialize and to promote the sustained support of the MOF. The iterative nature of the SHFS process is important, given the dynamic sociopolitical, economic, and technological contexts of LMICs and varied health system capacity, to ensure the strategy is internally consistent and directed towards achieving desired objectives (Box).

BOX The Five Objectives of a Surgical Health Care Financing Strategy

1. **Justification:** Establish reasons for why the ministries of health and finance should expand fiscal space for a national surgical, obstetric, and anesthesia plan (NSOAP), using investment case formation. (Step 1)
2. **Feasibility:** Quantify the funding amount or range of investment that could be allocated for an NSOAP, using fiscal space analysis. (Step 2)
3. **Positioning:** Determine how to frame an NSOAP when communicating with promising NSOAP funders, using stakeholder analysis. (Step 3)
4. **Efficiency:** Align financing of the NSOAP with broader health system financing, government budgeting process, and macro-fiscal constraints and opportunities. (Step 3)
5. **Accountability:** Promote transparency around NSOAP financial commitment and improve public financial management on surgical health care spending. (Step 3)

National health spending decisions are products of political decisions and processes. Any effective strategy to increase public expenditure for health must consider the political and budgetary processes in the country. The SHFS is no exception and should consider how the formal budget process is organized to secure public funding and align the NSOAP process with health fiscal constraints. An important question to ask is: why do governments prioritize some health matters over others and incorporate them within national health budgets? The answer to this question requires an understanding of the budgeting process and the key players and associated structures involved in budgetary decision-making within the government. Although there is a formal budgeting process, the way decisions are made is not always transparent. There are both internal and external factors relating to a country’s political system that dynamically influence the budgeting process and the health agenda. Internal factors might include, among others, party factions,^49^ the extent of corruption,^50^^,^^51^ or the degree of decentralization,^52^^–^^54^ and external factors could include special interests at the individual (populist leaders), group (new political parties), and institutional levels (donor countries and bilateral entities within the global health system).

#### Step 1: Justification of the Investment for an NSOAP and Expanded Surgical Health Care by Developing an Investment Case

Many NSOAP processes are initiated by motivated clinicians—champions of surgical health care—who are acutely aware of the need to improve surgical health care from their clinical experience but who lack the systematic evidence and authority within government needed to garner the full and sustained support of the MOH and MOF.

The objective of Step 1 is to develop the reasons that enable surgical health care to receive attention and sustained support, which are requisites for funding from governments and their development partners. Governments prioritize funding decisions based on investment cases, which outline the arguments in support of funding a particular sector. A high-level investment case for surgical health care should be developed (and reiterated as the NSOAP process evolves) to expand fiscal space and generate funding. The investment case consists of 4 elements: (1) cost: total investment expense and time frame; (2) health system: expected changes in health system inputs and outputs; (3) impact: improvement in population health outcomes and health system performance; and (4) broader government goals: positive spillover effects in other government sectors and priorities.

A high-level investment case for surgical health care should be developed to expand fiscal space and generate funding for surgical health care.

The NSOAP process supports the collection of data for investment case formation. We provide a summary of potential indicators and data sources for each of these 4 elements to develop a credible investment case (Table 2). The situation analysis, in particular, as the first step of the NSAOP process, determines current gaps in care using a validated surgical health care assessment tool.^55^ Countries with fully costed NSOAPs can use the estimated costs, together with current surgical expenditure data, to construct a rigorous investment case.

**TABLE 2.** Indicators to Formulate a Surgical Health Care Investment Case

**Components of an Investment Case**	**Indicators**	**Potential Data Sources**
Cost	All costed activities needed to deliver the proposed reform to improve surgical health care	NSOAP
Health systems	Percentage of population with 2-hour access to timely SOA care SOA providers per 100,000 population Hospital bed density ICU bed density	MOH World Bank WHO Primary data collection Research studies
Impact	Life expectancy Maternal mortality Mortality of children aged younger than 5 years Post-cesarean section mortality rate Post-operative mortality rate Percentage of population at risk of catastrophic expenditure Percentage of population at risk of impoverishing expenditure from SOA health care	MOH Hospital registries World Bank Data World Development Indicators
Broader government goals and spillover effects	Promote economic growth Reduce inequality and poverty Promote social cohesion Attain universal health coverage Achieve regional geopolitical dominance Promote national glory Reduce fossil fuel dependence	Country statistical office Country development plan UNDP WHO World Bank IMF

Abbreviations: ICU, intensive care unit; IMF, International Monetary Fund; MOH, Ministry of Health; NSOAP, national surgical, obstetric, and anesthesia plan; SOA, surgical, obstetric, and anesthesia; UNDP, United Nations Development Programme; WHO, World Health Organization.

Finally, it is important to examine the political reasons why it might be advantageous for a government to invest in surgical health care and to anticipate and harness political windows of opportunity to elevate its political priority. A government will not allocate substantial funding to these services if there is excessive political or economic risk or if the investment is not well aligned with its overarching vision and goals as well as the national interest. Analyzing the principal political drivers of government within its particular context will help determine how best to position surgical health care for investment. For instance, if reducing youth unemployment and poverty are major priorities, demonstrating how surgical health care could promote a healthy workforce and thereby contribute to human capital needed for economic growth^15^ could enhance the persuasiveness of the investment case. A more in-depth assessment, as the NSOAP evolves, will reveal the acute (immediate risk factors contributing to surgical disease) and systemic or structural (e.g., the political and economic roots of violence and trauma surgical need in South African townships) causes of youth disability and mortality due to surgery. Such granular detail and data will refine the overall positioning approach.

#### Step 2: Quantification of a Feasible Investment Range for an NSOAP and Expansion of Surgical Health Care

Step 2 aims to quantify the feasible investment range for an NSOAP and identify likely sources of funding using the fiscal space approach.^41^ Fiscal space refers to the ability of a government to increase public spending without compromising macroeconomic stability.^56^ Policymakers use fiscal space analysis to map funding sources that enable governments to increase public spending (the Supplement Box provides a detailed discussion of each fiscal space and how each pillar could be used independently or in combination to increase resource mobilization). Fiscal space analysis has been applied specifically to the health sector^41^ to examine health sector spending^57^^,^^58^; however, it has not been applied to identify sources of additional funding for surgical health care.

We propose the use of a modified fiscal space analysis,^59^ which entails examining 6 funding sources that can be used to create fiscal space in a country, namely: (1) macroeconomic conditions that influence level of government revenue; (2) reprioritization of government budget; (3) reprioritization of health sector–specific resources to cost-effective interventions; (4) health system efficiency in the use of existing resources; (5) external funding such as grants or loans; and (6) innovative financing.

The objective of Step 2 is to quantify a feasible investment range for an NSOAP based on appraising the likelihood of resource attainment from each fiscal space. Prospects of fiscal space expansion can be grouped into high, moderate, and low. All fiscal space sources directly or indirectly impact the government health budget, which, in turn, affects public spending on health. Table 3 provides a summary of the key metrics to assess each source. An in-depth analysis of each source allows the quantification, target setting, and monitoring of the progress of securing funding from each source as the NSOAP is developed and implemented. Though the private sector is not included in fiscal space analysis, exploring private sector investment could provide alternative pathways to expand fiscal space.

The objective of Step 2 is to quantify a feasible investment range for an NSOAP based on appraising the likelihood of resource attainment from each fiscal space.

**TABLE 3.** Fiscal Space Metrics

**Fiscal Space**	**Measurement**
Macroeconomic conditions	Projected economic growth Tax collection capabilities Level of inflation (consumer price index) Unemployment rate Balance of payments Debt: gross domestic product ratio Government taxes on externalities influencing population health
Reprioritization of government budget	Health budget as proportion of total government budget Annualized rate of change in health budget
Reprioritization of health sector–specific resources to cost-effective interventions	Health disparities by region and income level Population coverage with primary health care services
Efficiency in the application of existing health resources	Control of corruption index Health outcomes for health spending per capita Surgical volume of 1,000 per 100,000 population
External funding sources	Development assistance for health: total health expenditure ratio Development assistance for health: government health expenditure ratio
Innovative financing	Innovative financing: total health expenditure ratio Innovative financing: government health expenditure ratio

#### Step 3: Engagement of Selected Funders

Step 3 focuses on securing funding by identifying the most promising funders and then engaging each with a targeted investment case tailored to their interests. Funders refer to both the individuals and institutions that make resource allocation decisions, which influence each funding source. We provide a list of the relevant funders within each fiscal space source (Supplement Table 2).

**FIGURE 2:**
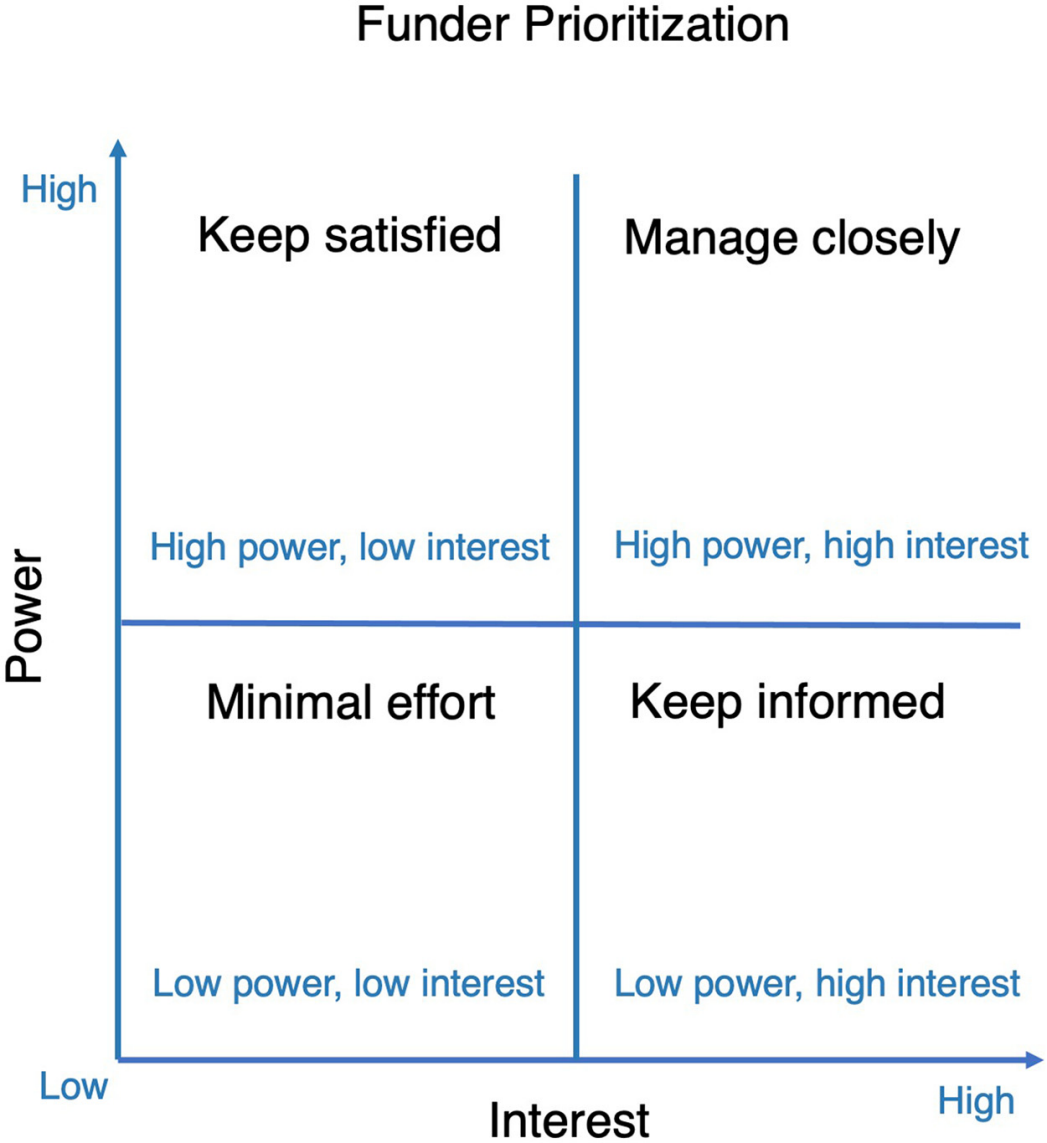
Stakeholder Analysis and Mapping^a^ ^a^Adapted from Mendelow.^61^

A stakeholder analysis determines which funders to prioritize and establishes how to engage them optimally. This is achieved by conducting a detailed analysis of each funder and by developing a targeted investment case based on the funder’s unique interests and preferences. Funders could be analyzed in terms of their influence and interest. Influence (or power) refers to the power of a player and the degree to which it affects funding decisions. Interest relates to assessing a funder’s level of commitment to funding the development and expansion of surgical services compared to other priorities. An analysis of these characteristics—influence and interest—can help determine which funders to prioritize and engage (Figure 2).^60^ Additionally, trust, or reliability, may also be included in the analysis. Trust considers how dependable a funder might be in terms of continued funding of an NSOAP over the long term (Supplement Figure).

A targeted investment case consists of aligning the reasons developed in Step 1 to the needs, interests, and goals of each of the prioritized funders. Funders will likely require different levels of engagement before, during, and after the NSOAP development process, depending on their level of influence, interest, and reliability. For example, stakeholders such as the MOF may not need to be involved in every step of the NSOAP development process. However, it will be advantageous to engage them before embarking on the NSOAP process, both during NSOAP costing and during the budget consultatory process.

## PART 3: A UNIFYING FRAMEWORK AND POLICY CONSIDERATIONS

### SHFS and NSOAP Alignment

**FIGURE 3:**
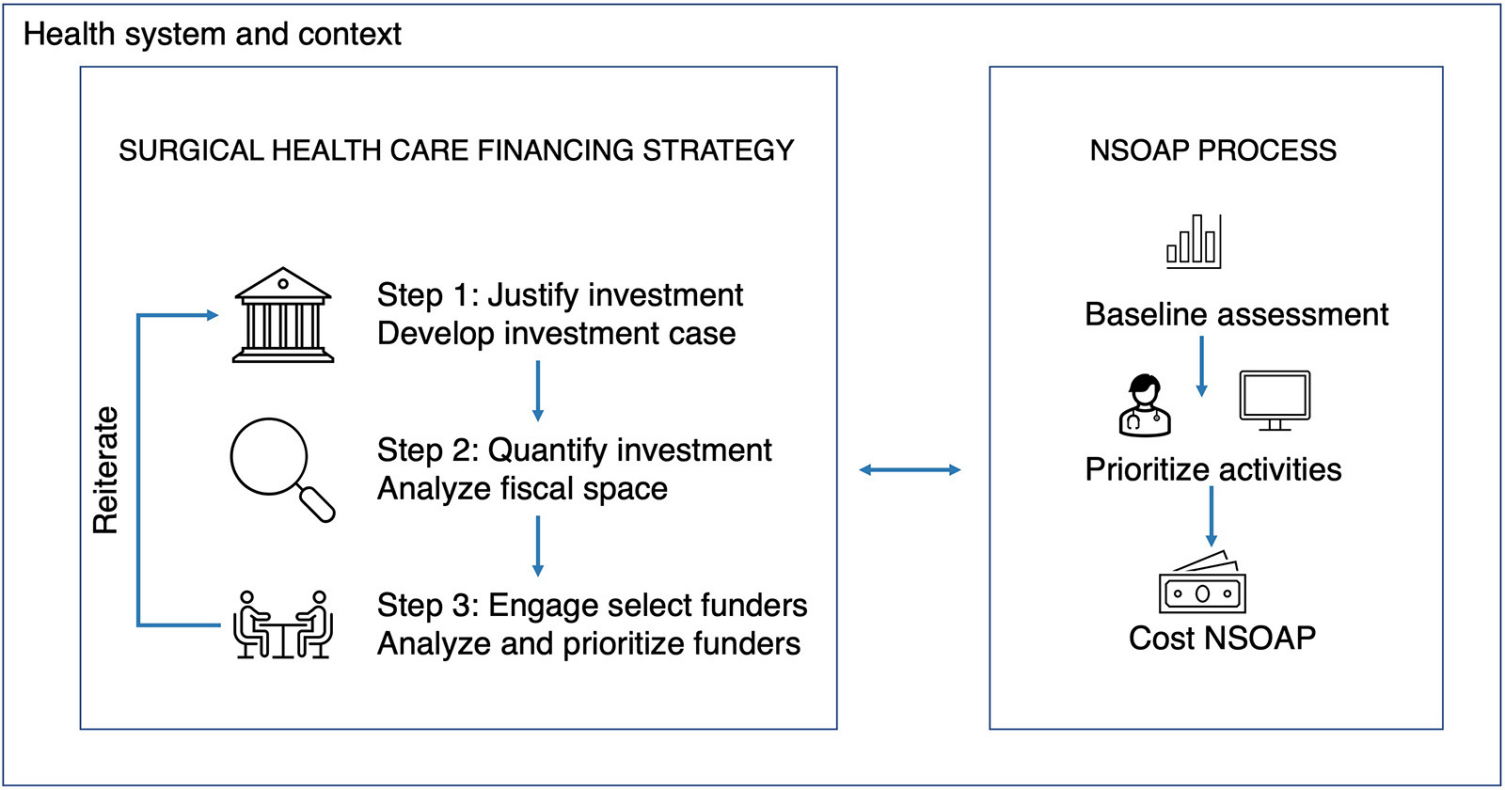
Aligning the Surgical Health Care Financing Strategy With the NSOAP Process Abbreviation: NSOAP, national surgical, obstetric, and anesthesia plan.

The purpose of the SHFS is to secure funding for policy instruments such as NSOAPs that are designed to improve surgical health care and implement an essential surgical package determined by each country as part of UHC. Figure 3 represents the SHFS process in parallel relation to the NSOAP within a broader context. They are both processes that depend on assessing and engaging with people and institutions in a context of dynamic change. The context consists of the health system and broader changes in the economy, political system, technology, and culture, which influence the pressure exerted on the health system, what is demanded of it, and its subsequent level of performance. These changes in context influence the priorities and interests of funders. Consider, for example, how the COVID-19 pandemic has led to a worldwide reprioritization of health expenditures. Within this context of change, the SHFS approach aims to impart intentionality and structure to the funding process. It is a dynamic and iterative process: both the SHFS and NSOAP are continuously modified as they evolve to respond to this dynamic complexity inherent in health systems.^61^ For example, when a country’s economic prospects change (e.g., an unfavorable economic outlook), the SHFS and NSOAP must adapt to this new pressure exerted upon the country’s health system. As a response, the SHFS might adapt to rely more upon innovative financing or improving the efficiency of health expenditures than on budget reprioritization to identify more appropriate sources to fund an NSOAP. A constrained SHFS, in turn, may require limiting the NSOAP to fewer costed activities and a limited essential surgical package as part of UHC to produce a feasible plan, given these changes.

The SHFS and NSOAP are both continuously modified as they evolve to respond to the dynamic complexity inherent in health systems.

### Policy Considerations for Financing Surgical Health Care

What are the funding scenarios that governments will face in LMICs? Certain SHFS options (Supplement Table 3) are more feasible than others, depending on the country’s income level, economic performance, capability to collect taxes, scale and scope of the tax base, health system demands, and the degree to which citizens and civil society shape the health and national agenda.

#### Domestic Source Predominant SHFS

Upper-middle-income countries, such as China, Mexico, South Africa, Thailand, and Turkey, are likely to find fiscal space from domestic sources. With conducive macroeconomic conditions, more extensive and diversified economies, considerable budgets, and greater capability to collect taxes from a substantial tax base, these countries are more likely to generate fiscal space from domestic sources, including efforts to unlock funding through improving efficiency and curbing corruption. These countries are likely to have the MOH institutional capacity to conduct an SHFS through established institutional processes. Since these countries often have highly organized private health care sectors and possess more favorable investor confidence, it may be suitable to explore private sources of funding.

#### Mixed-Resource SHFS

Lower-middle-income countries like Bolivia, Cameroon, Pakistan, and Vietnam could use a combination of external and domestic sources. This group of countries will likely receive small amounts of DAH relative to their health budgets or compared to that for LICs. However, these countries could mobilize funding from the domestic fiscus, provided there is continued macroeconomic growth, sufficient bureaucratic efficiency to collect taxes, and an enabling political environment in which the MOF increases the prioritization of the health budget.

#### External Source Predominant SHFS

External sources will likely be most applicable to LICs such as Haiti, Liberia, Malawi, Nepal, and Yemen, which are less likely to expand fiscal space for surgical health care from domestic sources due to less favorable macroeconomic conditions combined with a narrow tax base and low ability to collect taxes, amidst numerous and profound developmental challenges beyond the health sector. With smaller health budgets, LICs have less space to optimize the allocation of funding to surgical health care. LICs qualify for DAH, which usually comprises a substantial portion of their total health expenditure. Developing innovative financing mechanisms that pool funds from various sources and innovative instruments could also help to expand fiscal space. Since DAH is dependent on the stability of the global economic system, sustainability will be a critical element of the SHFS.

## CONCLUSION

The primary aim of the SHFS is to enable the MOH to develop a coherent strategy to fund surgical health services and expand care as part of broader efforts to achieve UHC. Where possible, the SHFS should be used to help align NSOAP financing with broader health system and government budgeting processes. It will also help to promote responsibility, accountability, and transparency about financial commitments around funding NSOAPs and other policies designed to improve surgical health care through the implementation of an essential surgical package via UHC.

The SHFS is not meant to be prescriptive but should be applied flexibly by adapting to changing country contexts. Though the SHFS has been used to identify funding sources for surgical health care through NSOAPs, the approach—justify investment, quantify investment, and engage selected funders—could be applied to other health priority areas for which funding is needed.

To our knowledge, this is the first time a systematic approach to financing has been proposed for global surgical health care. In the LMIC context, a coordinated governance approach to NSOAPs—led by the MOH with the support of the MOF and guided by health providers and patients through their professional associations and civil society organizations, respectively—is necessary to develop and follow through on plans and policies to strengthen surgical health care.

## Supplementary Material

GHSP-D-21-00295-supplement.pdf

## Notes

**Peer Reviewed**

**First published online:** May 2, 2023.

## References

[1] Meara JG Leather AJM Hagander L et al. Global Surgery 2030: evidence and solutions for achieving health, welfare, and economic development. Lancet. 2015;386(9993):569–624. 10.1016/S0140-6736(15)60160-X. 25924834

[2] Alkire BC Raykar NP Shrime MG et al. Global access to surgical care: a modelling study. Lancet Glob Health. 2015;3(6):e316–e323. 10.1016/S2214-109X(15)70115-4. 25926087 PMC4820251

[3] Bollyky TJ. Health without wealth: the worrying paradox of modern medical miracles. Foreign Affairs. September 26, 2018. Accessed March 30, 2023. https://www.foreignaffairs.com/articles/2018-09-26/health-without-wealth

[4] Hay SI Abajobir AA Abate KH et al.; GBD 2016 DALYs and HALE Collaborators. Global, regional, and national disability-adjusted life-years (DALYs) for 333 diseases and injuries and healthy life expectancy (HALE) for 195 countries and territories, 1990–2016: a systematic analysis for the Global Burden of Disease Study 2016. Lancet. 2017;390(10100):1260–1344. 10.1016/S0140-6736(17)32130-X. 28919118 PMC5605707

[5] Fox M. Climate change: what does it mean for the future of surgery? Bulletin of the American College of Surgeons. September 1, 2019. Accessed March 30, 2023. https://bulletin.facs.org/2019/09/climate-change-what-does-it-mean-for-the-future-of-surgery

[6] Ademuyiwa AO Bekele A Berhea AB et al. COVID-19 Preparedness within the surgical, obstetric, and anesthetic ecosystem in sub-Saharan Africa. Ann Surg. 2020;272(1):e9–e13. 10.1097/SLA.0000000000003964. 32301806 PMC7188046

[7] Peters AW Chawla KS Turnbull ZA. Transforming ORs into ICUs. N Engl J Med. 2020;382(19):e52. 10.1056/NEJMc2010853. 32329973 PMC7207079

[8] Shrime MG Dare AJ Alkire BC O’Neill K Meara JG. Catastrophic expenditure to pay for surgery worldwide: a modelling study. Lancet Glob Health. 2015;3(Suppl 2):S38–S44. 10.1016/S2214-109X(15)70085-9. 25926319 PMC4428601

[9] Gutnik L Yamey G Riviello R Meara JG Dare AJ Shrime MG. Financial contributions to global surgery: an analysis of 160 international charitable organizations. Springerplus. 2016;5(1):1558. 10.1186/s40064-016-3046-z. 27652131 PMC5021658

[10] GBD Compare. *The Lancet*. Accessed March 30, 2023. https://www.thelancet.com/lancet/visualisations/gbd-compare

[11] Shrime MG Bickler SW Alkire BC Mock C. Global burden of surgical disease: an estimation from the provider perspective. Lancet Glob Health. 2015;3(Suppl 2):S8–S9. 10.1016/S2214-109X(14)70384-5. 25926322

[12] Gawande A Debas HT Donkor P Jamison DT Kruk ME Mock CN. *Disease Control Priorities.* 3rd ed. World Bank Group; 2015. 26740991

[13] Alkire BC Shrime MG Dare AJ Vincent JR Meara JG. Global economic consequences of selected surgical diseases: a modelling study. Lancet Glob Health. 2015;3(Suppl 2):S21–S27. 10.1016/S2214-109X(15)70088-4. 25926317 PMC4884437

[14] Nepogodiev D Martin J Biccard B et al.; National Institute for Health Research Global Health Research Unit on Global Surgery. Global burden of postoperative death. Lancet. 2019;393(10170):401. 10.1016/S0140-6736(18)33139-8. 30722955

[15] Jumbam DT Reddy CL Makasa E et al. Investing in surgery: a value proposition for African leaders. Lancet. 2020;396(10243):7–9. 10.1016/S0140-6736(20)30482-7. 32622399 PMC7332255

[16] Sustainable Development Goals. UNDP. Accessed March 30, 2023. https://www.undp.org/sustainable-development-goals

[17] United Nations General Assembly. Political Declaration of the High-level Meeting on Universal Health Coverage: “Universal Health Coverage: Moving Together to Build a Healthier World.” September 23, 2019. Accessed March 30, 2023. https://www.un.org/pga/73/wp-content/uploads/sites/53/2019/05/UHC-Political-Declaration-zero-draft.pdf

[18] World Health Assembly, 68. *WHA 68.15: Strengthening Emergency and Essential Surgical Care and Anaesthesia as a Component of Universal Health Coverage*. World Health Organization; 2015. Accessed March 30, 2023. https://apps.who.int/gb/ebwha/pdf_files/WHA68/A68_R15-en.pdf 10.1007/s00268-015-3153-y 26239773

[19] World Health Organization (WHO). *Seventieth World Health Assembly: Resolutions and Decisions*. WHO; 2017. Accessed March 30, 2023. https://apps.who.int/gb/ebwha/pdf_files/WHA70-REC1/A70_2017_REC1-en.pdf

[20] Reddy CL Vervoort D Meara JG Atun R. Surgery and universal health coverage: designing an essential package for surgical care expansion and scale-up. J Glob Health. 2020;10(2):020341. 10.7189/jogh.10.020349. 33110540 PMC7562729

[21] Piot P Barré-Sinoussi F Karim QA Karim SSA Beyrer C. Appeal to global donors to save the Treatment Action Campaign. Lancet. 2014;384(9959):e62. 10.1016/S0140-6736(14)62045-6. 25479693

[22] Shawar YR Shiffman J Spiegel DA. Generation of political priority for global surgery: a qualitative policy analysis. Lancet Glob Health. 2015;3(8):e487–e495. 10.1016/S2214-109X(15)00098-4. 26187491

[23] Shiffman J. Four challenges that global health networks face. Int J Health Policy Manag. 2017;6(4):183–189. 10.15171/ijhpm.2017.14. 28812801 PMC5384980

[24] Jumbam DT Durnwald L Ayala R Kanmounye US. The role of non-governmental organizations in advancing the global surgery and anesthesia goals. J Public Health Emerg. 2020;4(0):18. 10.21037/jphe-2020-gs-07

[25] Albutt K Sonderman K Citron I et al. Healthcare leaders develop strategies for expanding national surgical, obstetric, and anaesthesia plans in WHO AFRO and EMRO regions. World J Surg. 2019;43(2):360–367. 10.1007/s00268-018-4819-z. 30298283

[26] Peters AW Roa L Rwamasirabo E et al. National surgical, obstetric, and anesthesia plans supporting the vision of universal health coverage. Glob Health Sci Pract. 2020;8(1):1–9. 10.9745/GHSP-D-19-00314. 32234839 PMC7108944

[27] Citron I Sonderman K Subi L Meara JG. Making a case for national surgery, obstetric, and anesthesia plans. Article in English and French. Can J Anaesth. 2019;66(3):263–271. 10.1007/s12630-018-01269-5. 30539367

[28] Citron I Jumbam D Dahm J et al. Towards equitable surgical systems: development and outcomes of a national surgical, obstetric and anaesthesia plan in Tanzania. BMJ Glob Health. 2019;4(2):e001282. 10.1136/bmjgh-2018-001282. 31139445 PMC6509614

[29] Burssa D Teshome A Iverson K et al. Safe surgery for all: early lessons from implementing a national government-driven surgical plan in Ethiopia. World J Surg. 2017;41(12):3038–3045. 10.1007/s00268-017-4271-5. 29030677

[30] Jumbam DT Reddy CL Roa L Meara JG. How much does it cost to scale up surgical systems in low-income and middle-income countries? BMJ Glob Health. 2019;4(4):e001779. 10.1136/bmjgh-2019-001779. 31478016 PMC6703298

[31] South African Development Community (SADC) Secretariat. *Media Statement - Joint Meeting of SADC Ministers of Health 2018*. SADC; 2018. Accessed March 30, 2023. https://web.archive.org/web/20210810215235/https://www.sadc.int/files/3315/4169/8409/Media_Statement_-_Joint_Meeting_of_SADC_Ministers_of_Health_and_those_responsible_for_HIV_and_AIDS_.pdf

[32] Reddy CL Makasa EM Biccard B et al. Surgery as a component of universal healthcare: where is South Africa? S Afr Med J. 2019;109(9):624–625. 10.7196/SAMJ.2019.v109i9.14233. 31635583

[33] *Thirteenth Pacific Health Ministers Meeting: Outcome of the Thirteenth Pacific Health Ministers Meeting, Tahiti, French Polynesia, 6–8 August 2019*. World Health Organization; 2019. Accessed March 30, 2023. https://apps.who.int/iris/handle/10665/351713

[34] World Health Organization (WHO). *Global Spending on Health: A World in Transition*. WHO; 2019. Accessed March 30, 2023. https://www.who.int/publications/i/item/WHO-HIS-HGF-HFWorkingPaper-19.4

[35] Dieleman J Campbell M Chapin A et al.; Global Burden of Disease Health Financing Collaborator Network. Evolution and patterns of global health financing 1995–2014: development assistance for health, and government, prepaid private, and out-of-pocket health spending in 184 countries. Lancet. 2017;389(10083):1981–2004. 10.1016/S0140-6736(17)30874-7. 28433256 PMC5440770

[36] Chang AY Cowling K Micah AE et al.; Global Burden of Disease Health Financing Collaborator Network. Past, present, and future of global health financing: a review of development assistance, government, out-of-pocket, and other private spending on health for 195 countries, 1995–2050. Lancet. 2019;393(10187):2233–2260. 10.1016/S0140-6736(19)30841-4. 31030984 PMC6548764

[37] Chen S Kuhn M Prettner K Bloom DE. The macroeconomic burden of noncommunicable diseases in the United States: estimates and projections. PLoS One. 2018;13(11):e0206702. 10.1371/journal.pone.0206702. 30383802 PMC6211719

[38] Costly noncommunicable diseases on rise in developing world. Harvard T.H. Chan School of Public Health News. June 21, 2011. Accessed March 30, 2023. https://www.hsph.harvard.edu/news/hsph-in-the-news/global-health-noncommunicable-diseases-bloom/

[39] Cutler D. How will COVID-19 affect the health care economy? JAMA Health Forum. 2020;1(4):e200419. 10.1001/jamahealthforum.2020.0419. 36218612

[40] Tandon A Cashin C. *Assessing Public Expenditure on Health from a Fiscal Space Perspective*. HNP Discussion Paper No. 56053. World Bank; 2010. Accessed March 30, 2023. https://documents1.worldbank.org/curated/en/333671468330890417/pdf/560530WP0Box341penditureFiscalSpace.pdf

[41] Dieleman JL Micah AE Murray CJL. Global health spending and development assistance for health. JAMA. 2019;321(21):2073–2074. 10.1001/jama.2019.3687. 31021368

[42] Atun R Silva S Ncube M Vassall A. Innovative financing for HIV response in sub–Saharan Africa. J Glob Health. 2016;6(1):010407. 10.7189/jogh.06.010407. 27231543 PMC4871060

[43] Lu C Schneider MT Gubbins P Leach-Kemon K Jamison D Murray CJL. Public financing of health in developing countries: a cross-national systematic analysis. Lancet. 2010;375(9723):1375–1387. 10.1016/S0140-6736(10)60233-4. 20381856

[44] Andrews M Cangiano M Cole N De Renzio P Krause P Seligmann R. *This Is Public Financial Management.* CID Working Paper No. 285. Center for International Development at Harvard University; 2014. Accessed March 30, 2023. https://www.hks.harvard.edu/centers/cid/publications/faculty-working-papers/pfm

[45] Gargasson JL Salomé B. The Role of Innovative Financing Mechanisms for Health. World Health Report (2010) Background Paper No. 12. World Health Organization; 2010. Accessed March 30, 2023. https://cdn.who.int/media/docs/default-source/health-financing/technical-briefs-background-papers/innovativebp12final.pdf

[46] Dieleman JL Yamey G Johnson EK Graves CM Haakenstad A Meara JG. Tracking global expenditures on surgery: gaps in knowledge hinder progress. Lancet Glob Health. 2015;3(Suppl 2):S2–S4. 10.1016/S2214-109X(15)70075-6. 25926316

[47] Financing Global Health | IHME Viz Hub. Accessed March 30, 2023. http://vizhub.healthdata.org/fgh

[48] Ritchie H. What do people die from? Our World in Data. February 14, 2018. Accessed March 30, 2023. https://ourworldindata.org/what-does-the-world-die-from

[49] Reddy T. The Congress Party Model: South Africa’s African National Congress (ANC) and India’s Indian National Congress (INC) as dominant parties. Afr Asian Stud. 2005;4(3):271–300. 10.1163/156920905774270493

[50] Lewis M. Governance and Corruption in Public Health Care Systems. Center for Global Development Working Paper No. 78. Center for Global Development; 2006. 10.2139/ssrn.984046

[51] Rispel LC de Jager P Fonn S. Exploring corruption in the South African health sector. Health Policy Plan. 2016;31(2):239–249. 10.1093/heapol/czv047. 26104821

[52] Bossert TJ Beauvais JC. Decentralization of health systems in Ghana, Zambia, Uganda and the Philippines: a comparative analysis of decision space. Health Policy Plan. 2002;17(1):14–31. 10.1093/heapol/17.1.14. 11861583

[53] Bossert TJ Larrañaga O Giedion U Arbelaez JJ Bowser DM. Decentralization and equity of resource allocation: evidence from Colombia and Chile. Bull World Health Organ. 2003;81(2):95–100. 12751417 PMC2572397

[54] Bossert TJ Mitchell AD Janjua MA. Improving health system performance in a decentralized health system: capacity building in Pakistan. Health Syst Reform. 2015;1(4):276–284. 10.1080/23288604.2015.1056330. 31519095

[55] National surgical, obstetric, and anesthesia planning. Harvard Medical School Program in Global Surgery and Social Change. Accessed March 30, 2023. https://www.pgssc.org/national-surgical-planning

[56] Heller PS. Understanding Fiscal Space. IMF Policy Discussion Paper No. 05/4. International Monetary Fund; 2005. Accessed March 30, 2023. https://www.imf.org/external/pubs/ft/pdp/2005/pdp04.pdf

[57] Barroy H Sparkes S Dale E. Assessing Fiscal Space for Health in Low and Middle Income Countries: A Review of the Evidence. Health Financing Working Paper No. 3. World Health Organization; 2016. Accessed March 30, 2023. https://apps.who.int/iris/handle/10665/251904

[58] Barroy H Kutzin J Tandon A et al. Assessing fiscal space for health in the SDG era: a different story. Health Syst Reform. 2018;4(1):4–7. 10.1080/23288604.2017.1395503

[59] Reddy CL Peters AW Jumbam DT et al. Innovative financing to fund surgical systems and expand surgical care in low-income and middle-income countries. BMJ Glob Health. 2020;5(6):e002375. 10.1136/bmjgh-2020-002375. 32546586 PMC7299051

[60] Mendelow AL. Environmental scanning - the impact of the stakeholder concept. In: *ICIS 1981 Proceedings.* International Conference on Information Systems; 1981:20. Accessed April 3, 2023. https://aisel.aisnet.org/cgi/viewcontent.cgi?article=1009&context=icis1981

[61] Atun R. Health systems, systems thinking and innovation. Health Policy Plan. 2012;27(Suppl 4):iv4–iv8. 10.1093/heapol/czs088. 23014152

