# Supplementary material for: A Financing Strategy to Expand Surgical Health Care
Source: Glob Health Sci Pract. 2023 Jun 21;11(3):e2100295. doi: 10.9745/GHSP-D-21-00295 (PMC10285720; doi:10.9745/GHSP-D-21-00295)
Supplement: GHSP-D-21-00295-supplement.pdf [file GHSP-D-21-00295-supplement.pdf]

Supplement to: Reddy CL, Jumbam DT, Meara JG, Makasa EM, Atun R. A financing strategy to expand surgical health care. *Glob Health Sci Pract*. 2023;11(3):e2100295. https://doi.org/10.9745/GHSP-D-21-00295

Supplement Table S1. Comparative costing of NSOAPs in LMICs

Country	Implementation time (years)	NSP total cost (US$)	Current Health Expenditure per capita (US$)	NSP cost/year per capita (US$)
Zambia	5	314,160,747	1509.80	3.62
Tanzania	7	597,042,037	936.33	1.51
Rwanda	6	69,735,072	748.39	0.94
Nigeria	5	16,768,118,788	1968.56	17.12

Source: Adapted from Jumbam DT, Reddy CL, Roa L, Meara JG. How much does it cost to scale up surgical systems in low-income and middle-income countries? *BMJ Glob Health* 2019; **4**: e001779.

Supplement to: Reddy CL, Jumbam DT, Meara JG, Makasa EM, Atun R. A financing strategy to expand surgical health care. *Glob Health Sci Pract*. 2023;11(3):e2100295. https://doi.org/10.9745/GHSP-D-21-00295

Supplement Box: Understanding the sources of funding for fiscal space and how countries expand public spending

Macroeconomic conditions

The state of a country's economy influences health fiscal policy. In general, the degree to which a country's economy grows, the greater extent a government may spend on its policies. Increased government revenue can lead to increased domestic funding for health, even if the level of prioritisation (proportion of the total budget allocated to the health sector) remains constant. In Turkey, for example, the sustained economic growth from 2000-2008, allowed the government to increase public sector funding for health at an annualised average growth rate of 9.1%, paving the way for UHC scale-up.(1) In India, increases in GDP was found to be the most significant predictor of increased government health spending.(2) In studies that conduct comparative country assessments of fiscal space, economic growth is the most frequently cited source of fiscal space expansion.(3) Though the MoH has limited influence over policies to stimulate economic growth, macroeconomic conditions are a major driver of health sector spending and must be taken into account when assessing the likelihood of increasing government spending on surgical care.

Government budget reprioritisation

Reprioritising government budget such that a higher proportion is allocated to health, expands fiscal space. The Abuja Declaration recommended that sub-Saharan African Nations increase their health budgets to at least 15% of the national GDP to strengthen their health systems;(4) one sub-Saharan African countries attained this target.(5,6) An analysis of costs required to implement NSOAPs reveals that between 0.13% and 0.87% of GDP per capita or 0.57% or 13.2% of the annual government budget is needed to implement NSOAPs (Table 1).(7) While budget reprioritisation is a political process, an evidence-based investment case, together with a compelling framing of surgical care, could be used to make a persuasive case for budgetary reform.

Health-sector specific resources

The health budget may be increased by generating additional resources from earmarked taxes and establishing social insurance. Widespread examples include introducing excise *sin taxes* on specific goods with health externalities such as tobacco,(8) alcohol,(9) sugar,(10,11) and reducing government subsidies.(12) The International Monetary Fund estimates that global energy subsidies (on a post-tax basis) amounted to $US 2 trillion, roughly 2.9% of the global GDP, or 8.5% of total government revenue. These subsidies could be earmarked for the health sector to combat the externalities of energy production and climate change on health.

Establishing or expanding social insurance is another way to expand fiscal space. Organising different health-specific sources from government revenue (tax and non-tax), private and voluntary sectors into a pooled fund could also expand the fiscal space for surgical services through UHC.

Efficiency of existing resources

This pillar refers to the ability of a government to translate its financial resources into health services that produce improvements in population health. Efforts to optimise efficiency, it is estimated, could increase health expenditure by up to US$8 per capita.(13) Efficiency has both allocative and technical aspects. Allocative efficiency refers to funding an optimal mix of health system inputs that will produce the most considerable population health benefit. Technical efficiency is concerned with producing the maximum output at the least cost. The health system of France, one could argue, is more technically efficient than that of the United States because it achieves superior health outcomes while spending less per capita. Assessing the proximal and distal causes of both technical and allocative inefficiency of available resources can be used to limit wasteful expenditure and expand fiscal space. Finally, rent-seeking behaviour is a substantial factor that influences the efficiency of public expenditure, including health.(14,15) The drivers are numerous but include government subsidies, price controls, low civil service wages, natural resource endowments,(16) and government procurement.(17) Though challenging to quantify the scope of corruption, of the estimated $US 7 trillion spent annually on healthcare, 10-25% is lost indirectly through corrupt practices.(18)

External sources

External sources form a substantial component of financing many LIC health systems. In 2014, DAH financed 35.7% of health spending in LICs.(19) Major funders such as the World Bank, Bill & Melinda Gates Foundation, and several bilateral agencies have contributed to the growth of DAH. USAID, for example, channelled $27.2 million to The Human Resources for Health programme in Rwanda, which enabled the rapid training of physicians needed to deliver Universal Health Coverage.(20) When considering DAH as a potential source of fiscal space expansion for NSOAPs, its limitations should also be taken into account. First, DAH is a volatile and less sustainable form of health systems financing compared to domestic funding as it is influenced by global economic shocks such as the global 2008 recession and COVID-19 pandemic. Second, it is essential to consider how DAH affects domestic resource allocation. Evidence suggests that for every dollar year-to-year increase in DAH, recipient governments tend to remove $0.60 from the health sector.(21) This fungibility suggests that increased DAH may not lead to fiscal space expansion if governments reduce their contribution to the health sector as a result of DAH.

Innovative financing sources

Innovative financing has been used in the global health arena to help fund health priorities for which there is inadequate funding at the national level. The Global Fund, an example of an innovative financing mechanism established in 2002, focuses on HIV/AIDS, Tuberculosis, and Malaria. In South Africa, the Global Fund disbursed US$ 973,395,559 to help provide antiretrovirals to 4.35 million people with HIV/AIDS through the governments National HIV/AIDS program(22). Other examples of innovative financing that have been taken to scale include the Global Alliance for Vaccines and Immunisation (GAVI), UNITAID, and the Children's Investment Fund Foundation (CIFF), which focus on vaccinations, malnutrition, and child health respectively. Innovating financing does not merely provide new sources of funding to healthcare. The distinguishing feature of innovative financing is that innovation occurs at multiple points involved in the production of a particular health service. Innovative mechanisms typically develop new sources of funding, pool numerous funding streams, channel through a single streamlined entity, allocate to high-impact health needs, and implement for performance and value such that innovation enhances health system gains.

Supplement to: Reddy CL, Jumbam DT, Meara JG, Makasa EM, Atun R. A financing strategy to expand surgical health care. *Glob Health Sci Pract*. 2023;11(3):e2100295. https://doi.org/10.9745/GHSP-D-21-00295

Supplement Table S2. Funders by Fiscal Space Source

Source of fiscal space	Funders
**Macroeconomic conditions**	- Ministry of Finance
**Reprioritisation of government budget**	- Minister of Health and cabinet members<br>- Minister of Finance and cabinet members<br>- Parliamentarians<br>- Other government officials (e.g. secretary to MoF)<br>- Civil society organisations<br>- Influential politicians<br>- Media outlets
**Health sector-specific resources**	- Minister of Health and cabinet members<br>- Minister of Finance and cabinet members<br>- Parliamentarians<br>- Other government officials<br>- Civil society organisations<br>- Influential politicians<br>- Media outlets
**Efficiency of existing resources**	- Minister of Health and cabinet members<br>- Finance and Accounts department of MoH<br>- Health Economists<br>- Parliament
**External resources**	- Bilateral organisations (e.g. USAID, DFID, JICA, KOICA)<br>- Multilateral organisations (e.g. WHO, World Bank, African Development Banks, Islamic Development Bank, UN)<br>- Private organisations (e.g. BMGF, Wellcome Trust)
**Innovative financing sources**	- Government

Source: Original

Supplement Table 3. Comparison of Policy Options to Finance Surgical Healthcare by SHFS Fiscal Space

SHFS fiscal space dominance	Country group	Pros	Cons
**(A) Domestic**	UMIC	- Predictable and sustainable<br>- Not driven by donor interests<br>- Promotes responsibility in investment<br>- Opportunity to improve spending efficiency	- Requires a clear political strategy to influence national health budget<br>- Competing priorities from other state sectors<br>- Dependent on sustained macroeconomic growth
**(B) External**	LIC	- Can leverage existing relationships with funders<br>- Opportunity to explore innovative mechanism and reduce aid dependence	- Not sustainable or reliable<br>- Sustains dependence<br>- Fungibility<br>- Conditionality and donor special interests
**(C) Mixed**	LMIC	- combination of A+B	- combination of A+B
**Innovative**	All	- Potential to mobilise untapped funding streams<br>- Funding linked to implementation performance<br>- Ability to pool funding at a supra-national level	- Complicated to establish<br>- Need to develop innovative instruments

Source: original

Supplement to: Reddy CL, Jumbam DT, Meara JG, Makasa EM, Atun R. A financing strategy to expand surgical health care. *Glob Health Sci Pract*. 2023;11(3):e2100295. https://doi.org/10.9745/GHSP-D-21-00295

Supplement Figure. Stakeholder Analysis

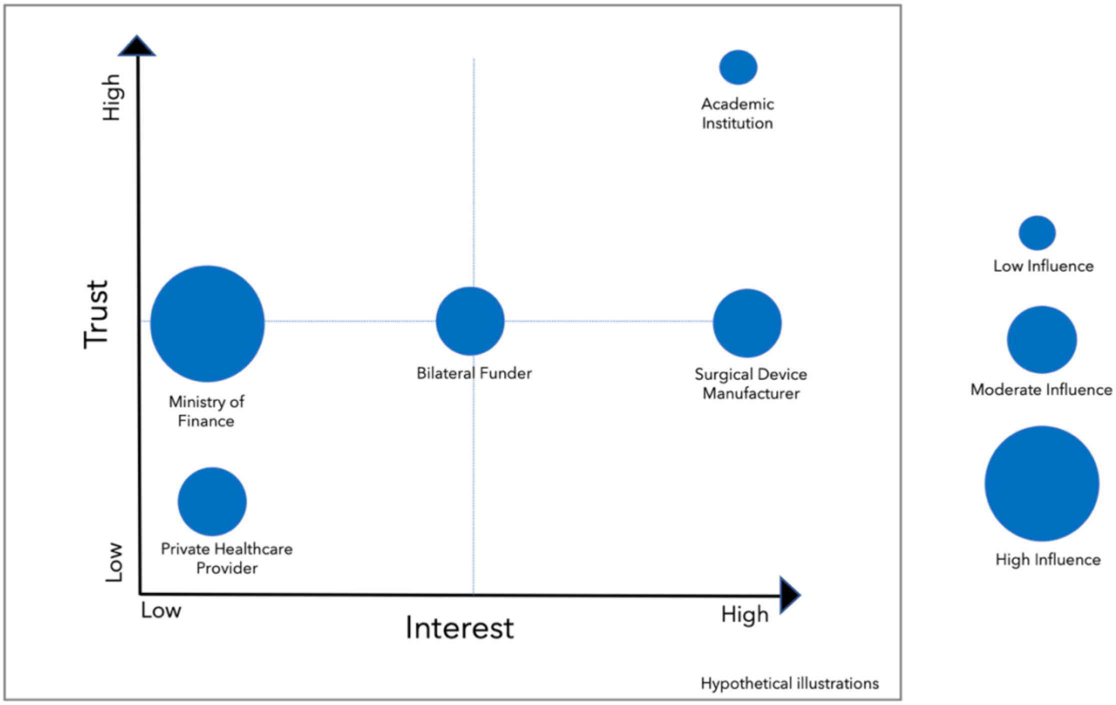

Source: Adapted from Adapted from A.L., Mendelow, A.L. (1981). Environmental Scanning - The Impact of the Stakeholder Concept, International Conference on Information Systems (ICSI) 1981 Proceedings

REFERENCES

1. Atun R, Aydin S, Chakraborty S, Sümer S, Aran M, Gürol I, et al. Universal health coverage in Turkey: Enhancement of equity. The Lancet. 2013;382(9886):65–99.

2. Behera DK, Dash U. Effects of economic growth towards government health financing of Indian states: an assessment from a fiscal space perspective. Journal of Asian Public Policy [Internet]. 2017 Nov 6 [cited 2020 Jan 16]; Available from: https://www.tandfonline.com/doi/abs/10.1080/17516234.2017.1396950

3. Barroy H, Sparkes S, Dale E, Mathonnat J. Can Low- and Middle-Income Countries Increase Domestic Fiscal Space for Health: A Mixed-Methods Approach to Assess Possible Sources of Expansion. Health Systems & Reform [Internet]. 2018 Jun 1 [cited 2020 Jan 16]; Available from: https://www.tandfonline.com/doi/abs/10.1080/23288604.2018.1441620

4. Abuja Declaration [Internet]. Africa Union; 2001. Available from: https://www.who.int/healthsystems/publications/abuja_report_aug_2011.pdf?ua=1

5. Micah AE, Chen CS, Zlavog BS, Hashimi G, Chapin A, Dieleman JL. Trends and drivers of government health spending in sub-Saharan Africa, 1995-2015. BMJ Glob Health. 2019;4(1):e001159.

6. WHO | The Abuja Declaration and the plan of action. An extract from the African Summit on Roll Back Malaria [Internet]. WHO. [cited 2016 Jul 14]. Available from: http://www.who.int/malaria/publications/atoz/whocdsrbm200346/en/

7. Jumbam DT, Reddy CL, Roa L, Meara JG. How much does it cost to scale up surgical systems in low-income and middle-income countries? BMJ Global Health [Internet]. 2019 Aug 1 [cited 2020 Jan 17];4(4). Available from: https://gh.bmj.com/content/4/4/e001779

8. Organisation mondiale de la santé. WHO report on the global tobacco epidemic, 2015: raising taxes on tobacco. 2015.

9. Sornpaisarn B. Resource tool on alcohol taxation and pricing policies. :108.

10. WHO commends South African parliament decision to pass tax bill on sugary drinks [Internet]. WHO | Regional Office for Africa. [cited 2019 Sep 21]. Available from: https://www.afro.who.int/news/who-commends-south-african-parliament-decision-pass-tax-bill-sugary-drinks

11. Taxes on sugary drinks: Why do it? [Internet]. The World Health Organization; 2017 [cited 2019 Nov 17]. Available from: https://apps.who.int/iris/bitstream/handle/10665/260253/WHO-NMH-PND-16.5Rev.1-eng.pdf?sequence=1

12. Jamison DT, Summers LH, Alleyne G, Arrow KJ, Berkley S, Binagwaho A, et al. Global health 2035: a world converging within a generation. The Lancet. 2013 Dec;382(9908):1898–955.

13. Barroy H, Sparkes S, Dale E. Assessing fiscal space for health in low and middle income countries: a review of the evidence. 2016;(3). Available from: http://www.wipo.int/amc/en/

14. Delavallade C. Corruption and distribution of public spending in developing countries. J Econ Finan. 2006 Jun;30(2):222–39.

15. Malyniak BS, Martyniuk OM, Kyrylenko OP. The Impact of Corruption on the Efficiency of Public Spending Across Countries with Different Levels of Democracy. Financial and credit activity: problems of theory and practice. 2019;1(28):290–301.

16. International Monetary Fund. Why Worry About Corruption? [Internet]. WASHINGTON, D.C.: INTERNATIONAL MONETARY FUND; 1997 [cited 2020 Apr 28]. (Economic Issues). Available from: http://elibrary.imf.org/view/IMF051/15223-9781557756350/15223-9781557756350/15223-9781557756350.xml

17. Preventing Corruption in Public Procurement [Internet]. Organization for Economic Cooperation and Development; 2016 [cited 2020 Apr 28]. Available from: https://www.oecd.org/gov/ethics/Corruption-Public-Procurement-Brochure.pdf

18. García PJ. Corruption in global health: the open secret. The Lancet. 2019 Dec;394(10214):2119–24.

19. Dieleman J, Campbell M, Chapin A, Eldrenkamp E, Fan VY, Haakenstad A, et al. Evolution and patterns of global health financing 1995–2014: development assistance for health, and government, prepaid private, and out-of-pocket health spending in 184 countries. The Lancet. 2017 May;389(10083):1981–2004.

20. Binagwaho A, Kyamanywa P, Farmer PE, Nuthulaganti T, Umubyeyi B, Nyemazi JP, et al. The Human Resources for Health Program in Rwanda — A New Partnership. N Engl J Med. 2013 Nov 21;369(21):2054–9.

21. Dieleman JL, Hanlon M. Measuring the Displacement and Replacement of Government Health Expenditure. Health Econ. 2014 Feb;23(2):129–40.

22. South Africa - Results - The Global Fund Data Explorer [Internet]. [cited 2019 Nov 17]. Available from: https://data.theglobalfund.org/investments/results/ZAF
